# An Imported Case of Afebrile *Plasmodium falciparum* Malaria Infection from Tanzania in a Returning Traveler to the Republic of Korea following an Earlier COVID-19 Infection

**DOI:** 10.3390/tropicalmed7040059

**Published:** 2022-04-08

**Authors:** Chaeryoung Lee, Sung Kwan Hong, Jong Hun Kim

**Affiliations:** Division of Infectious Diseases, Department of Internal Medicine, CHA Bundang Medical Center, CHA University, Seongnam 13496, Korea; folareta70@chamc.co.kr (C.L.); skhong@cha.ac.kr (S.K.H.)

**Keywords:** afebrile, *Plasmodium falciparum* malaria, COVID-19 infection

## Abstract

Malaria is well-known as one of the most common causes of fever among travelers returning from endemic areas such as tropical African countries. However, afebrile *Plasmodium falciparum* malaria has rarely been reported in a returning traveler with no prior history of malaria infection. Here, we report an imported case of afebrile *P. falciparum* malaria infection from Tanzania in a returning traveler to the Republic of Korea, following an earlier COVID-19 infection without previous history of malaria infection. Our case suggests the hypothesis that severe symptoms of *P. falciparum* malaria infection might be prevented by cross- immunity from previous COVID-19 infection.

## 1. Introduction

Malaria is well-known as one of the most common causes of fever among travelers returning from malaria-endemic areas such as tropical African countries [1]. In addition to fever, common symptoms of malaria infection are nonspecific, and may include chills, myalgias, diaphoresis, and headache [2]. In contrast, asymptomatic malaria infection can be found in certain individuals in malaria-endemic areas who acquired immunity against malaria from past malaria infections [3]. However, the population with little or no immunity against malaria, such as travelers with no previous history of malaria infection, would be vulnerable to malaria, with a higher risk of developing symptomatic malaria infection. Thus, afebrile *Plasmodium falciparum* malaria in a returning traveler with no prior history of malaria infection has rarely been reported. Here, we report an imported case of afebrile *P. falciparum* malaria infection from Tanzania in a returning traveler to the Republic of Korea following an earlier coronavirus disease 2019 (COVID-19) infection, without previous history of malaria infection.

## 2. Case Report

**Figure 1:**
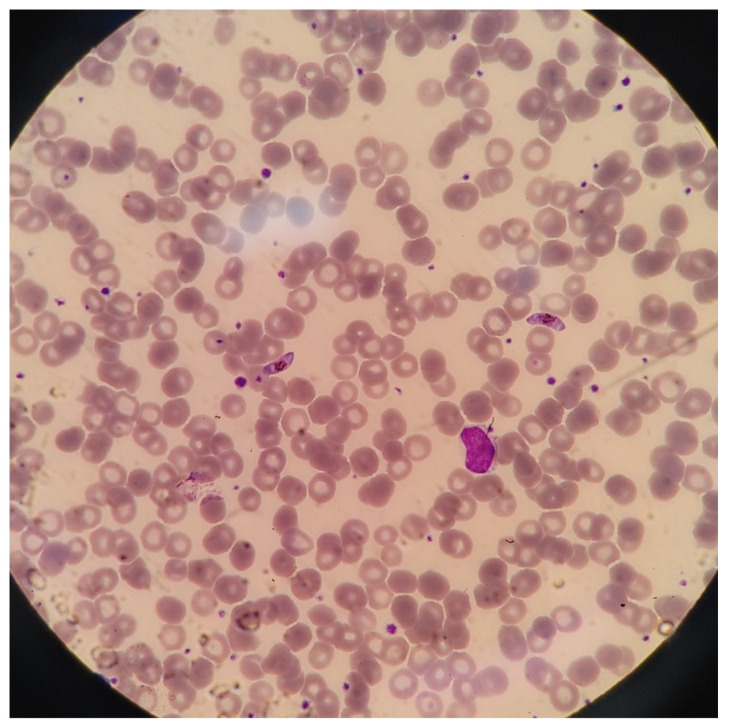
Peripheral smear for blood cell morphology showing *P. falciparum* gametocytes (Magnification, ×400, Wright stain).

A 63-year-old Korean man with hypertension and dyslipidemia had traveled back and forth between Korea and Tanzania since 2017. Although he had not taken malaria chemoprophylaxis medication, he had not previously been diagnosed with malaria infection. However, in January 2021, he experienced febrile illness while staying in Tanzania and underwent diagnostic tests, which were positive for COVID-19 and negative for malaria infection. Despite the lack of specific antiviral treatment, his medical condition improved, and he recovered after a month. However, he developed urinary symptoms of frequent urination with nocturia over the course of several months. Thus, he left Tanzania after confirmation of a negative COVID-19 test and arrived in Korea on 2 May 2021. After 14 days of mandatory quarantine period, he presented to the hospital on 17 May 2022. A stable condition was noted without other specific symptoms except for his urinary symptoms. He was afebrile, with a temperature of 36.8 °C. His blood pressure was 126/82 mm Hg, heartrate was 102 beats per minute, and respiratory rate was 18 breaths per minute with oxygen saturation of 100% in room air. The physical examination was unremarkable, without hepatosplenomegaly, jaundice, abdominal tenderness, and costovertebral angle tenderness. Laboratory examinations showed white blood cells (5.3 × 10^9^/L), platelets (294 × 10^9^/L), aspartate transaminase (33 IU/L), alanine transaminase (44 IU/L), total bilirubin (0.77 mg/dL), urine analysis (urine red blood cell < 1 per high-power field and urine white blood cell < 1 per high-power field), prothrombin time (11.3 s), and activated partial thromboplastin time (35.9 s), were within the normal ranges, except for mildly anemic hemoglobin (12.8 g/dL). The inflammatory marker of C-reactive protein was also unremarkable (0.2 mg/dL). However, atypical material suspicious for malaria gametocytes was found from the routine complete blood-count test result review process by a laboratory technician. For further investigation, a peripheral smear for blood cell morphology test was performed, which showed crescentic gametocytes, indicating *P. falciparum* malaria infection with parasitemia (952/µL) (Figure 1). Therefore, additional malaria polymerase chain reaction (PCR) tests (EONE Laboratories, Republic of Korea) were performed, and he was treated with atovaquone and proguanil for 3 days. His malaria PCR tests were positive for *P. falciparum* and negatives for *P. vivax*, *P. malariae*, and *P. ovale*. After completion of malaria treatment, he was diagnosed with overactive bladder and placed on naftopidil and mirabegron, which resulted in a subsequent improvement in his urinary symptoms. On an outpatient follow-up visit, he remained stable, and his follow-up peripheral smear for blood cell morphology test on 3 June 2021 was negative for malaria. 

## 3. Discussion

As endemic *P. falciparum* malaria has not been reported in Korea since 1945 [4], most Koreans born after 1945 with age < 75 years are likely to be considered *P. falciparum* malaria-naïve. Reported cases of *P. falciparum* malaria infection in Korea were classified as imported malaria infections [5]. Although our case patient had traveled back and forth between Korea and Tanzania since 2017, he was deemed naïve to *P. falciparum* malaria as he had not previously been diagnosed with malaria infection. Despite the likelihood of developing symptomatic *P. falciparum* malaria infection, as would be expected in the *P. falciparum* malaria-naïve individuals, such as our case patient, he was afebrile and did not present the typical features of *P. falciparum* malaria infection. The pathogenesis of asymptomatic *P. falciparum* malaria infection in our patient is not clearly understood; however, there are several factors to consider. First, our patient had COVID-19 infection caused by severe acute respiratory syndrome coronavirus 2 (SARS-CoV-2) in January 2021, approximately 4 months prior to the diagnosis of *P. falciparum* malaria infection. There have been reports of the common immunodominant epitopes between SARS-CoV-2 and *P. falciparum* [6,7]. These common immunodominant epitopes were found in the antigens that facilitate SARS-CoV-2 and *P. falciparum* infection of the host through invasion of the erythrocyte [6]. Furthermore, several other common epitopes with the capacity to induce the stimulation of CD8+ T-lymphocytes via the recognition of human leukocyte antigen (HLA)-A*02:01 were found [6]. As SARS-CoV-specific memory T cell responses were found to persist for up to 11 years [8], SARS-CoV-2-specific memory T cell responses are predicted to persist for a similar length of time [9]. Therefore, it can be assumed that the immune responses elicited by previous COVID-19 infection may prime lasting CD8+ T-lymphocytes that can recognize common immunodominant epitopes from subsequent *P. falciparum* malaria infection, leading to protection against the dissemination and progression of *P. falciparum* malaria infection. Second, SARS-CoV-2 is known to have various glycoproteins (GPs): membrane GPs, spike GPs, and GPs that have properties of hemagglutination [10,11,12]. Following the recovery of COVID-19 infection, antibodies against GPs such as spike GPs are produced [13]. In addition, various autoimmune antibodies were found to be produced after COVID-19 infection [14]. Interestingly, anti-plasmodial activity and protection against malaria by autoimmune responses from various autoimmune antibodies were reported [15]. Therefore, it could be hypothesized that diverse antibodies, including autoimmune antibodies and antibodies against GPs produced after COVID-19 infection, could offer protection against subsequent malaria infection or severity. Our case has some limitations; we did not examine immunologic effects to test our hypothesis. There was also the possibility of confounders, such as the presence of certain human leukocyte antigen alleles that might have potential effects on the development of symptoms of malaria infection since additional laboratory tests were not performed. Nonetheless, our case suggests that the complex constellation of immune processes following COVID-19 infection might play a protective role against subsequent *P. falciparum* malaria infection.

In summary, afebrile *P. falciparum* malaria infection in the individual from non-endemic areas is rare. However, our case suggests that severe symptoms of *P. falciparum* malaria infection might be prevented by cross-immunity from previous COVID-19 infection. With this case report, we would like to draw attention to the diagnostic challenge presented to clinicians by the atypical presentation of illness, possibly influenced by the current ongoing COVID-19 pandemic. Further studies, involving a larger number of patients, are needed to better define the pathogenesis of afebrile *P. falciparum* malaria infection following COVID-19 infection.

## Data Availability

The data presented in this study are available on request from the corresponding author.
